# Tyrosine Kinase Inhibitor Imatinib Mesylate Alters DMBA-Induced Early Onco/Suppressor Gene Expression with Tissue-Specificity in Mice

**DOI:** 10.1155/2019/8670398

**Published:** 2019-02-07

**Authors:** Péter Attila Gergely, Balázs Murnyák, János Bencze, Andrea Kurucz, Timea Varjas, Katalin Gombos, Tibor Hortobágyi

**Affiliations:** ^1^Institute of Forensic Medicine, University of Debrecen, 4012 Debrecen, Hungary; ^2^MTA-DE Cerebrovascular and Neurodegenerative Research Group, Department of Neurology, University of Debrecen, 4012 Debrecen, Hungary; ^3^Institute of Pathology, Faculty of Medicine, University of Debrecen, 4012 Debrecen, Hungary; ^4^Department of Pharmacology and Pharmacotherapy, University of Debrecen, 4012 Debrecen, Hungary; ^5^Department of Public Health Medicine, Medical School, University of Pécs, 7624 Pécs, Hungary; ^6^Department of Laboratory Medicine, Clinical Center, University of Pécs, 7624 Pécs, Hungary; ^7^Institute of Pathology, University of Szeged, 6725 Szeged, Hungary

## Abstract

Tyrosine kinases play crucial roles in cellular development and tumorigenesis. Tyrosine kinase inhibitors (TKIs) are effective and widely used drug molecules in targeted cancer therapies. Altered expressions of protooncogenes and tumor suppressor genes after DMBA (7,12-dimethylbenz[a]anthracene) treatment have been described as early markers of tumor induction; however their tissue-specific effects remain still unclear. Our study was aimed at examining the short-term possible antineoplastic and chemopreventive effects of a TKI compound (imatinib mesylate) on a DMBA-induced mouse tumor model. In addition, we also investigated the tissue-specific expressions of* Hras, Kras, Myc,* and* Trp53* genes in the brain, bone marrow, spleen, liver, abdominal lymph nodes, thymus, lungs, and kidneys, respectively. 24 hours after the imatinib mesylate injection, we observed significant* Kras* downregulation in the bone marrow and lung of the DMBA-treated mice. Moreover, the mRNA expression of* Myc *was also found to be decreased significantly in the spleen. Interestingly, while* Trp53* expression was significantly increased in the lung, it was decreased in the other tissues. However, there was also a tendency in the decreased* Myc *level in the bone marrow, brain, kidneys, lungs, and lymph nodes and in the decreased* Hras* level in the bone marrow, kidneys, and lungs, although no significant differences were observed. Our findings indicate rapid tissue-specific impact of imatinib mesylate on DMBA-induced gene expression* in vivo,* supporting the chemopreventive potential of imatinib mesylate in cancer.

## 1. Introduction

Protein kinases (PKs) play pivotal roles in cellular processes such as metabolism, proliferation, apoptosis, immune response, or nervous system functions. PKs regulate enzyme activity by phosphorylating cellular proteins [1] and their dysregulation may lead to pathological conditions, i.e., different types of cancers or inflammatory diseases. Therefore, PKs have become one of the most extensively investigated drug targets in the past two decades [2]. To date, the human PK gene family consists of 518 members and can be categorized into nine groups. Among them, tyrosine kinases (TKs)—and their inhibitor molecules—are the most promising targets of cancer studies [3]. TKs are classified as receptor and nonreceptor tyrosine kinases. Receptor tyrosine kinases (RTKs) are transmembrane proteins consisting of an extracellular ligand-binding domain and an intracellular kinase domain [4]. Nonreceptor tyrosine kinases can be found in the cytosol and nucleus or in the inner part of the plasma membrane, participating in the regulation of cell proliferation or differentiation [5]. The activation of TKs is under tight control. Their kinase activity is low in nonproliferating cells. On the contrary, TK expression is extremely increased in cancer cells, caused by ligand or receptor overexpression by various mechanisms [6–11].

Imatinib was the first small-molecule TKI that accomplished a remarkable clinical success in the treatment of chronic myeloid leukemia (CML). Imatinib mesylate inhibits the constitutively active BCR-Abl protein kinase that is responsible for the constant proliferation of myeloid cells [12]. Druker et al. reported that imatinib produced a 92-98% decrease in the number of colonies from BCR-Abl cells, while having minimal effect on normal cells [13]. Imatinib targets further protein kinases, including the stem cell factor receptor (c-kit) and the platelet-derived growth factor receptor (PDGFR), whose inhibition might have potential implications for the treatment of several malignancies [14]. Imatinib treatment is usually well-tolerated; however, side effects may develop, e.g., edema, nausea, skin rash or moderate myelosuppression [15]. Resistance to imatinib can occur within months or years after the beginning of the treatment. Several mechanisms of resistance have been discovered, categorized as BCR-Abl-dependent (like point mutation in the protein kinase domain of Abl, amplification, or overexpression of the gene) [16]) or independent (decreased drug uptake, increased efflux, or upregulation of secondary signal transduction pathway elements, such as Ras-Raf-MEK-ERK) [17]).

Other tyrosine kinase inhibitors include sunitinib for metastatic renal cell carcinoma [18], sorafenib for clear-cell renal carcinoma [19], gefitinib for advanced non-small cell lung cancer [20], erlotinib for the treatment of pancreatic cancer [21], lapatinib for women with advanced breast cancer [22], pazopanib for locally advanced or metastatic renal cell carcinoma [23], vandetanib for advanced non-small-cell lung cancer [24], and axitinib as a second line therapy for metastatic renal cell carcinoma [25]. This class of small-molecule drugs offers enormous promise for targeted management of malignant diseases. A growing body of evidence suggests that suppressing the secondary signal transduction pathway intensity by TKI-s might be promising target in antitumor therapy [26]. Oncogenes and tumor suppressor genes play essential roles in tumorigenesis. The ‘classical' mammalian RAS protooncogenes (*HRAS, KRAS, *and* NRAS*), the* MYC* protooncogene, and the tumor suppressor* TP53* gene are of great relevance in tumorigenesis. Ras proteins are small GTP-ase transcription factors that play a regulatory role in MAPK and PI3K secondary signal transduction pathways. Their disturbed functions result in cell proliferation and death [27]. Mutant Ras proteins are constitutively active, leading to uncontrolled cell proliferation, and can be associated with nearly one-third of human cancers such as pancreatic, epidermal, lung, colorectal cancers, or multiple myeloma [28].* Myc* is a member of the MYC oncogene family (*Myc, Mycn,* and* Mycl*) that encodes a phosphoprotein being able to transform cells through multiple pathways [29, 30]. Associated with almost 70% of human cancers [31],* Myc* is a master regulator of tumorigenesis and development through modulating the activity of genes in cell proliferation, apoptosis, tumor suppression, DNA repair, angiogenesis, and invasion [32]. P53 is the most extensively studied tumor suppressor protein, since its gene is mutated nearly in half of the human tumors [33]. The majority of mutations occur in the DNA-binding domain; however, mutations may be observed in every region of the human* TP53* gene [34, 35]. Two forms of* TP53* mutation exist: ‘loss-of-function' and ‘gain-of-function' mutations [36]. ‘Loss-of-function' mutations lead to loss of oncosuppressive activity, while ‘gain-of-function' mutations may result in numerous different effects including enhanced tumor cell invasion and motility [37], chemoresistance [38], proliferation [39], and enhanced cell survival [40].

In a previous study, we investigated the antineoplastic and chemopreventive properties of four tyrosine kinase molecules in the liver, lung, bone marrow, and kidney of a DMBA (7,12-dimethylbenz[a]anthracene) induced mouse preclinical tumor model by examining the expression of* Hras* and* Trp53* genes. DMBA is a widely used polycyclic aromatic hydrocarbon chemical carcinogen that initiates chemical carcinogenesis by inducing various oncogenic mutations resulting in lung tumor, squamous cell carcinoma, and vascular tumors (hemangiomas), as well as intestinal, mammary, uterine, or hematologic tumors [41, 42]. The results suggested that chalcone analogues, as intermediary compounds of the flavonoid biosynthetic pathway, and plant derivatives may possess potential chemopreventive effects [43].

In this study, we assessed the short-term tissue-specific effects of imatinib mesylate on the expression of* Hras, Kras, *and* Myc* and* Trp53* genes in the bone marrow, brain, kidneys, liver, lungs, lymph nodes, spleen, and thymus of DMBA-treated mice.

## 2. Materials and Methods

### 2.1. Experimental Animals

Six- to eight-week-old (25±5 g) conventionally raised NMRI inbred mice (n=12, 6 males and 6 females in each group) were involved in our study, which was approved by the Animal Experiment Committee of University of Pécs (BA 02/2000-16/2011). The mice were housed six animals per cage at an ambient temperature under a 12h:12h light:dark cycle with* ad libitum *access to chow food and water.

### 2.2. Treatment Group Assignment

**Figure 1:**
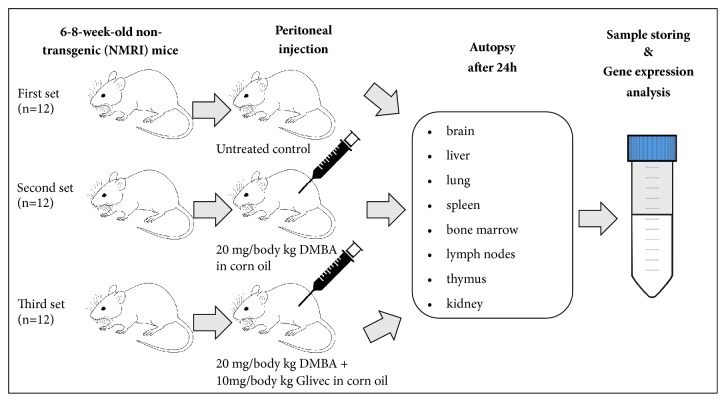
*Experimental design*. Six- to eight-week-old (n=36) conventionally raised NMRI inbred mice were divided randomly into three sets: the negative control group was i.p. treated with the vehicle (corn oil) (1st set, n=12), the positive control group (2nd set, n=12) was treated i.p. with a 20 mg/kg body weight dose DMBA (7,12-dimethylbenz[a]anthracene), and the experimental group (3rd set, n=12) was treated i.p. with 10 mg/kg imatinib mesylate and 20 mg/kg DMBA. Animals were autopsied 24 hours after treatment, and organs were dissected and stored for further analysis.

Three experimental sets were created for the experimental agents (Figure 1). The first set of animals was treated intraperitoneally (i.p.) with vehicle (corn oil) and served as a negative control group. The second set of mice (positive control) was treated i.p. with a 20 mg/kg dose of DMBA dissolved in corn oil (both compounds were purchased from Sigma Aldrich, Budapest, Hungary). In the third group (experimental set), animals were simultaneously treated i.p. with 10 mg/kg imatinib mesylate (4-[(4-methyl-1-piperazinyl)methyl]-N-[4-methyl-3-[[4-(3-pyridinyl)-2-pyrimidinyl]amino]-phenyl]benzamide methanesulfonate, Novartis Pharma GmbH product (Glivec), and 20 mg/kg DMBA dissolved in corn oil. Mice were sacrificed 24 hours after the injections, and organs (liver, spleen, kidney, lung, thymus, lymph node, bone marrow, and brain) were harvested and snap-frozen in liquid nitrogen and then stored at - 80°C for further use.

### 2.3. RNA Extraction

100 mg tissue samples of each organ from the respective groups were homogenized in MagNA Lyzer Green Beads tubes (Roche (Hungary) Ltd.) using the MagNA Lyzer instrument (Roche (Hungary) Ltd.). Total RNA was isolated from the tissue lysates using the EXTRAzol RNA extraction kit (Invitrogen Life Technologies Magyarország Kft). The RNA quality was assessed by absorption measurement at 260/280 nm (A260/A280 was >1.8).

### 2.4. Gene Expression Investigations

One-step PCR including reverse transcription and target amplification was performed using Kapa SYBR FAST One-step RTqPCR Kit (Kapa Biosystems) on a LightCycler 480 qPCR platform with a 96-well format. The specific primers (IDT) for mouse tumor suppressor genes (*Hras*, 5′-AATTGGGGGAGCAAGGACAT-3′); (*Kras*, 5′-TATCCTGCTTCCCATCAGTGTTC-3′); (*Myc*, 5′-GTTGTGCTGGTGAGTGGAGA-3′); (*Trp53*, 5′-CTTCACTTGGGCCTTCAAAA-3′) and for a housekeeping gene (*Gapdh*, 5′-CACATTGGGGGTAGGAACAC-3′) were used in the quantitative amplification.

RT-qPCR was initiated by 5 min. and 3 min. incubations at 42°C and 95°C, respectively, followed by 50 cycles (95°C for 10 s, 55°C for 20 s, and 72°C for 20 s) with a fluorescent reading taken at the end of each cycle. Each run was completed with a melting curve analysis (95°C for 5 s, 65°C for 60 s, and 97°C ∞) to confirm the specificity of amplification. Fluorescent values were calculated following the ΔΔCp method on Exor 4 software (Roche (Hungary) Ltd.) and gene expressions are reflected as relative quantification results.

### 2.5. Data Analysis

Statistical analyses were performed using R software (http://www.r-project.org) and SPSS 21.0 software (SPSS Inc., IL, USA). The differences in mRNA expression levels were calculated using a two-tailed Student's t-test and were considered to be significant when p<0.05. Gene-gene interaction networks to demonstrate the relationship between genes in different organs/experimental sets were generated by the GeneMania Cytoscape 3.4.0 application. Physical, coexpression, and gene-gene interactions were evaluated [44]. Heat map was constructed using Gene-E version 3.0.204 (http://www.broadinstitute.org/cancer/software/GENE-E/index.html).

## 3. Results

### 3.1. Gene Expression

**Figure 2:**
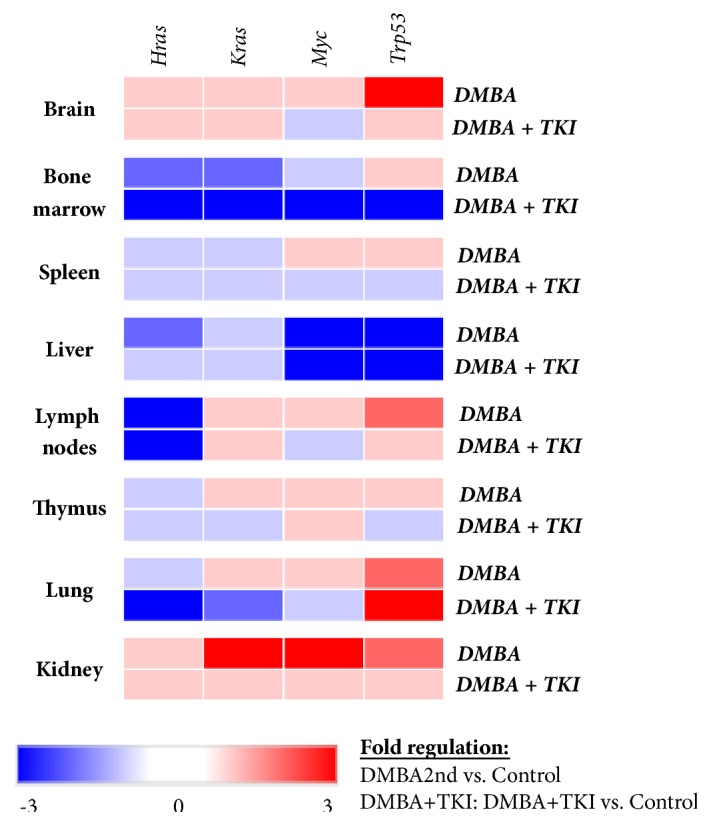
*Heat map of gene expression patterns compared to the negative control*. Blue boxes represent negative (down) regulation, while red boxes indicate positive (up)regulation of the gene expression.

**Figure 3:**
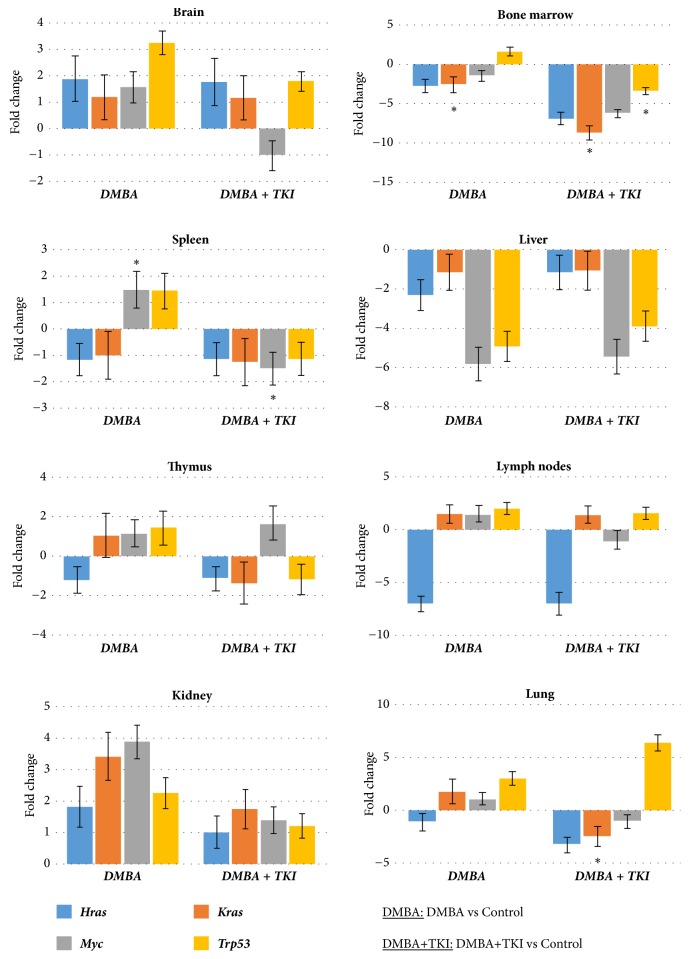
*Gene expression pattern of experimental groups in different organs*. *∗* p<0,05.

Gene expression patterns of the three experimental sets are shown on Figures 2 and 3. Importantly, we found no gender-specific differences in the gene expression patterns.

#### 3.1.1. Bone Marrow

In the bone marrow, DMBA injection decreased the expressions of* Hras, Kras,* and* Myc, *respectively, and increased* Trp53 *expression. DMBA+imatinib mesylate administration further decreased the* Hras, Kras,* and* Myc* expressions. Compared to the negative control, significantly lower* Kras* expressions were found in the second* (p<0.05)* and third sets of mice* (p<0.05).* The combined treatment also decreased the expression of the tumor suppressor* Trp53 *to a significant extent (*p<0.05*), first (control) versus third (DMBA + imatinib mesylate) set.

#### 3.1.2. Brain

Compared to the negative controls, DMBA administration resulted in increased gene expressions in the brain; however, these changes were found to be nonsignificant. Combined administration of DMBA and imatinib mesylate decreased the expressions of the studied genes; however, these alterations were not significant either.

#### 3.1.3. Kidney

DMBA increased the expressions of the* Hras, Kras,* and* Myc, *respectively, and the expression of the* Trp53*, as well. The simultaneous administration of DMBA and TKI reduced the expression of all the investigated genes.

#### 3.1.4. Liver

In the liver, DMBA administration lowered the expressions of* Hras, Kras, Myc,* and* Trp53*, respectively. As a result of the combined DMBA+TKI administration, the decrease in the expression of these genes became reduced.

#### 3.1.5. Lung

In the lung, mRNA expressions of the* Kras (p<0.05)*,* Myc,* and* Trp53 *genes were increased, while the* Hras *expression was decreased following the DMBA injection. Simultaneous treatment with DMBA and TKI led to decreased the expression of protooncogenes (*Hras, Kras,* and* Myc*) and increased* Trp53 *mRNA levels.

#### 3.1.6. Lymph Nodes

In the lymphoid tissues, DMBA decreased the* Hras* expression and increased the* Kras* and* Trp53 *expressions, that remained unchanged after the combined administration with DMBA+TKI. However, the expression of* Myc* was increased by DMBA and decreased as a result of DMBA+TKI combination. However, this change in mRNA expression was not statistically significant.

#### 3.1.7. Spleen

*Hras* and* Kras* gene expressions were decreased after DMBA injection, although they did not change after DMBA+TKI administration. In turn, DMBA induced increased expressions of* Myc* (*p<0.05*) and decreased* Trp53* expressions after treatment (DMBA+TKI).

#### 3.1.8. Thymus

In the thymus, DMBA increased the expressions of* Kras, Myc* and* Trp53*, respectively, while decreasing the* Hras expression*. As a result of combined administration of DMBA+imatinib mesylate, the expressions of* Kras* and* Trp53* were found to be reduced compared to the negative control. Additionally, the expression of* Myc* showed an increase, while the expression of* Hras* remained unaltered after the combined injections.

### 3.2. Gene Network

**Figure 4:**
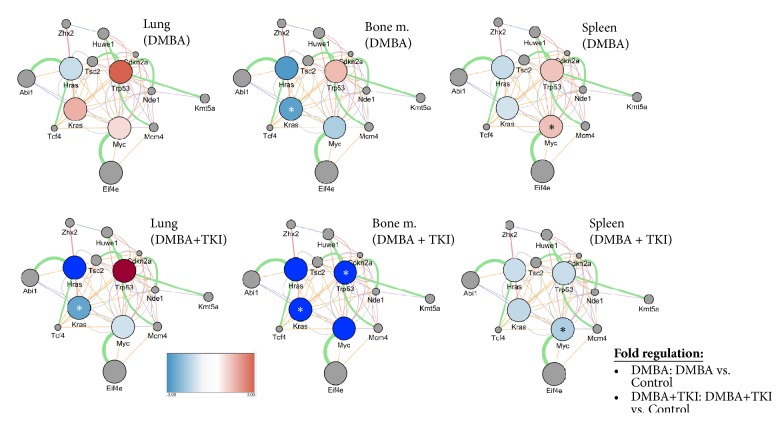
*A gene-gene interaction network presenting the correlation among the fold regulation of Kras, Hras, Myc, and Trp53 genes in the represented organs and their predicted interactions with 10 functionally related genes*. The 10 correlated genes were obtained using the GeneMania application of Cytoscape; level of significance: *∗* (p < 0.05).

Figure 4 shows the fold regulation of gene expressions in selected organs and their predicted interactions among the different regulatory genes. We observed significant alterations in gene expressions in the bone marrow, lung, and spleen. Our network analysis revealed that* Hras, Kras,* and* Myc* protooncogenes and* Trp53* tumor suppressor gene have extensive connections to other regulatory genes.* Zhx2* (also known as* RAF*) is a homodimeric transcription factor that belongs to the zinc fingers and homeoboxes gene family [45],* Abi1 *(abl interactor 1) is an adaptor protein that facilitates several signal transduction pathways, regulates actin polymerization and cytoskeleton remodeling, and therefore has a role in cell proliferation [46].* Tcf4* (transcription factor 4) is essential for neuronal development [47], and* Tsc2 *(TSC complex subunit 2) gene codes a tumor suppressor protein (tuberin), mutation of which (together with mutation of hamartin, coded by* Tsc1*) causes tuberous sclerosis complex [48].* Huwe1* encodes an E3 ubiquitin ligase protein that is responsible for ubiquitination and degradation of the antiapoptotic protein Mcl1 (myeloid cell leukemia sequence 1 (Bcl2-related)) [49].* Cdkn2a *(cyclin dependent kinase inhibitor 2a) is an important tumor suppressor gene, having at least three alternative spliced variants that code two CDK4 inhibitors and one p53 stabilizer protein, therefore playing a pivotal role in cell cycle G1 control [50].* Nde1* (nudE neurodevelopment protein 1) gene codes a protein that has essential role in microtubule organization, mitosis, and neuronal migration, mutation of which can be associated with lissencephaly [51].* Kmt5a *(lysine methyltransferase 5a) codes a protein that is a transcriptional repressor and is important for cell proliferation and chromatin condensation [52].* Mcm4* (minichromosome maintenance complex component 4) gene codes a protein that is highly conserved and important for initiation of eukaryotic genome replication [53].* Eif4e* (eukaryotic translation initiation factor 4E) functions as a protooncogene; its product helps the initiation of translation [54].

## 4. Discussion

Several studies have demonstrated the role of tyrosine kinases in human diseases [55, 56]. Consequently, tyrosine kinases have become one of the main areas of pharmacological experiments intended to develop targeted drugs [57]. Protein tyrosine kinase inhibitors are small molecules that are able to diffuse through the cell membrane targeting cytoplasmic kinases or the intracellular domain of receptor tyrosine kinases. TKIs are currently booming and are widely used in cancer cure either in the form of monotherapy or in combination with other chemotherapeutic agents [58].

In our present study, we investigated the potential chemopreventive effect of imatinib mesylate that is the first small-molecule tyrosine kinase inhibitor used in CML and gastrointestinal stromal tumor (GIST) [59, 60]. To date, our study is among the first ones to examine the possible preventive effect of imatinib mesylate by studying the alterations in DMBA-induced gene expression levels and trying to put the results into the gene network of different protooncogenes (*Hras, Kras,* and* Myc*) and a tumor suppressor gene (*Trp53*) in a short-term experiment. The outcomes shown here suggest that imatinib mesylate might have a possible mitigating role in diseases beyond CML and GIST.

Major results of the present study include that short-term DMBA treatment (i) elevated the expression of all the three protooncogenes (*Hras, Kras,* and* Myc*) in the brain and kidneys; (ii) increased the level of* Kras* and* Myc* in the lung, lymph nodes and thymus; (iii) increased the expression of the tumor suppressor gene* Trp53 *that can be considered an adaptive physiologic countermeasure in response to a chemical carcinogen. These phenomena have been previously described by several investigations, concluding that DMBA is a potent inducer of chemical carcinogenesis and can be used for studying different types of malignant tumors. DMBA is a polyaromatic hydrocarbon similar to hydrocarbons to which humans can be exposed. DMBA causes point mutations in protooncogenes like* Hras* that is common in human carcinomas [61]. In the bone marrow and liver, DMBA decreased the expression level of* Hras, Kras,* and* Myc*. This observation might be explained by the fact that DMBA is a carcinogenesis inducer, and it is usually applied simultaneously with a carcinogenesis promoter, e.g., 12-O-tetradecanoylphorbol 13-acetate (TPA) [62]. Therefore, in case of the bone marrow and liver, DMBA might not be enough for complete tumorigenesis. In the spleen, the elevated level of* Myc* was the only prominent and significant alteration in the gene expression pattern. Several studies have elucidated the role of* Myc* in tumorigenesis. Probably the best-established association is that nearly every case of Burkitt's lymphoma involves rearrangement and therefore overexpression of* Myc* with a regulatory element of immunoglobulin heavy or light chains or other nonrandom somatic mutations of the gene [63, 64]. The results of the aforementioned studies correlate with our finding of elevated expression of* Myc* in the spleen and lymph nodes as a consequence of DMBA treatment. The increased expression of the examined four genes gain more importance in the context of their extensive gene network.* Zhx2* (also known as* RAF*) has previously been associated with Hodgkin lymphoma [65] and hepatocellular carcinoma [66];* Abi1 *(abl interactor 1) has a role in colorectal carcinoma development and invasion [67] and also in neuroblastoma propagation [68]. Aberrant function of* Tcf4* (transcription factor 4) has been reported in glioblastoma [69] and in colorectal tumors [70].* Tsc2 *(TSC complex subunit 2) gene codes a tumor suppressor protein (tuberin), mutation of which have been associated with tumors in the brain, lungs, kidneys, skin, heart, uterus, and eyes [71, 72].* Huwe1* encodes an E3 ubiquitin ligase protein that is required for the development of colorectal carcinoma and ovarian tumors [73, 74].* Cdkn2a *(cyclin dependent kinase inhibitor 2a) is an important tumor suppressor gene predisposing to several tumors, e.g., urothelial carcinoma, hereditary melanoma, pancreas cancer, or non-small-cell lung cancer [75–77].* Nde1* (nudE neurodevelopment protein 1) gene codes a protein that has essential role in microtubule organization and mitosis, and recent studies have elucidated its potential role in acute or chronic myeloid leukemia [78, 79].* Mcm4* (minichromosome maintenance complex component 4) has been reported to be upregulated in ovarian cancer, skin cancer, or esophageal carcinoma [80–82].* Eif4e* (eukaryotic translation initiation factor 4E) functions as a protooncogene; its product has been suggested to regulate expression of proteins that are crucial for cell cycle progression, cell survival, and motility. A growing body of evidence implicates this translational factor in cell transformation, tumorigenesis, or tumor progression, e.g., in case of prostate cancer, lymphomas, CML, or lung cancers [83].

As it is suggested by the extensive gene network of the examined genes, cancer development involves more than one transforming events and the interaction of several oncogenes and tumor suppressor genes. This network and series of events offers numerous opportunities to effectively influence the process of tumorigenesis.

In the lungs, the expression of protooncogenes (*Hras, Kras,* and* Myc*) and their connections to other genes coding transcription factors or cell proliferation regulators (e.g.,* Tcf4, Abi1,* and* Zhx2)* prominently decreased as a result of the short-term combined DMBA+TKI treatment, while the expression of* Trp53 *gene increased. Comparing to the negative control, the decrease in* Kras* expression was significant.

In the bone marrow, DMBA+TKI combined treatment significantly decreased the expression and gene interactions of the* Kras* and* Trp53*.

DMBA+TKI treatment could significantly decrease the DMBA-induced increase in the expression and gene interactions of* Myc* protooncogene. The expression of the tumor suppressor* Trp53* also decreased following the combined treatment; however, this decrease was not significant.

Outcomes of our short-term experiment suggest that protein tyrosine kinase inhibitor treatment (imatinib mesylate) simultaneously administered with the chemical carcinogen, DMBA, might have an impact on the expression pattern of the examined protooncogenes (*Hras, Kras,* and* Myc)* and tumor suppressor gene (*Trp53*), therefore on the tumorigenesis, controlled by these genes.

Imatinib mesylate is a well-known small-molecule inhibitor of tyrosine kinases. In our study, this drug was able to decrease significantly the expression of* Kras* oncogene in the bone marrow and in the lung, as well as the expression of* Myc* oncogene in the spleen. Additionally,* Myc* mRNA expressions were tended to be lowered in the bone marrow, brain, kidneys, lungs, and lymph nodes and we also observed tendencies in the* Hras* mRNA expressions to be decreased in the bone marrow, kidneys, and lungs, although these changes were not statistically significant. The reduced expression of these oncogenes may be attributed to the kinase inhibitor effect of imatinib mesylate, as described by other recent studies. Among others, Lorri Puil et al. reported that BCR-Abl was able to activate Ras signaling in CML, by creating a direct link between Grb2 and mSos1 that are responsible for the conversion of inactive GDP-bound form of Ras into the active, GTP-bound form. Therefore, inhibiting BCR-Abl kinase activity may downregulate Ras signaling in CML [84].

Besides Ras signaling, BCR-Abl kinase can indirectly activate* Myc* either through the Janus-activated kinase 2 (JAK2) pathway [85] or by the mitogen-activated protein kinase (MAPK) pathway [86]. It is tempting to speculate that imatinib might have decreased the expression of* Myc* well before its DMBA-induced overexpression. Callahan R. et al. revealed that imatinib mesylate was able to induce complete regression of mammary tumor and restore lobuloalveolar development and lactation by inhibiting Notch4 and* Myc* signaling, which result also support the idea of therapeutic potential of imatinib mesylate, other than CML and GIST [87].

PDGF isoforms and their receptors (PDGFRs) are considered as prototypes of growth factors and receptor tyrosine kinases for more than 25 years. They are essential for normal gastrulation and cranial, neuronal, cardiac, pulmonary, intestinal, gonadal, hematological, skin, renal, and skeletal development, as well as for hematopoiesis, through the secondary signal transduction pathway, including activation of Ras and the downstream Raf and MAPK cascades [88]. However, overexpression or mutational events in the PGDFR gene may drive tumor development and progression [89]. Recent studies have elucidated the role of PDGFRs in the evolution of different nervous system tumors, i.e., glioblastoma [90], ependymoma [91], meningioma [92], and schwannoma (in which PDGFR mutation is usually associated with c-kit overactivation [93]). In addition to brain tumors, the role of mutant PDGFR has numerously been emphasized in other malignant diseases, like dermatofibrosarcoma protuberans [94], gastrointestinal stromal tumor (GIST) [95], osteosarcoma [96], alveolar rhabdomyosarcoma [97], chronic myeloid leukemia (CML) [98], prostate cancer [99], liver cancer [100], non-small-cell lung cancer [101], and colorectal cancer [102] and in breast cancer [103]. There have been numerous attempts to inhibit the activity of PDGFRs, including tyrosine kinase inhibitors, like imatinib or sorafenib, and also several antibodies targeting the different PDGF isoforms or the receptors themselves to prevent their activation. In general, antibodies are much more specific therapeutic tools; however, their administration is expensive and sometimes inconvenient. Tyrosine kinase inhibitors are not specific; they have the potential to inhibit more kinases—and in this way have more adverse effects—as imatinib is able to inhibit PDGFRs, Abl kinases, and the stem cell receptor c-kit, but in cancer treatment, it can be advantageous to target more than one component of tumorigenesis [89].

*Kras, Hras,* and* Myc* are the executive elements of numerous oncogenic pathways, so they can be favorable to inhibit a common point of tumorigenesis by one molecule. p53 is the best characterized tumor suppressor protein, as it is able to induce cell cycle arrest or cell death in response to hypoxia and incorrigible genetic mutations, while mutations of* TP53* gene have been associated with more that 50% of human tumors [104]. There is growing evidence that these mutations are ‘loss-of-function' mutations; however, missense mutations may result in simultaneous gain of functions that have usually detrimental effect to the cell [105]. Numerous studies have reported that mutant p53 played a key role in tumor development, progression, and invasion of several cancer types, e.g., in case of breast cancer [106], lung cancer [107], colorectal cancer [108], different brain tumors, and gastric adenocarcinoma [109]. In our present study, short-term imatinib mesylate treatment administered simultaneously with DMBA resulted in a prominent increase in the* Trp53* expression in the lung, while decreasing it in all the other tissues. These data indicate a possible ‘gain-of-function' mutation in the gene of the tumor suppressor p53 protein and that imatinib mesylate attempted to decrease the level of this aberrant protein.

Based on our recent and previous findings we suggest that imatinib mesylate is a promising chemotherapeutic agent for prevention and management of several malignant tumors by decreasing the mRNA expression of the protooncogenes and the mutant* Trp53 *gene.

## 5. Conclusion

The outcomes of the present study demonstrate that imatinib mesylate decreases the mRNA expressions of* Hras, Kras, Myc,* and* Trp53* genes in certain organs after 24 hours of a single dose of TKI treatment in a DMBA-induced mouse tumor model. These results suggest its preventive and curative roles in malignant diseases.

## Data Availability

The experimental analysis data used to support the findings of this study are available from the corresponding author upon request.
